# Population Persistence and Soil Microbial Communities of a Serpentine Endemic Plant Outside Its Historic Elevation Range

**DOI:** 10.1002/ece3.71629

**Published:** 2025-06-21

**Authors:** Courtney Collins, Devin Dinwiddie, Nuttapon Pombubpa, Krista McGuire, Marko J. Spasojevic

**Affiliations:** ^1^ Biodiversity Research Centre The University of British Columbia Vancouver BC Canada; ^2^ Institute of Arctic and Alpine Research University of Colorado Boulder Boulder Colorado USA; ^3^ Department of Biology University of Oregon Eugene Oregon USA; ^4^ Department of Microbiology, Faculty of Science Chulalongkorn University Bangkok Thailand; ^5^ Department of Microbiology & Plant Pathology University of California Riverside Riverside California USA; ^6^ Department of Evolution, Ecology, and Organismal Biology University of California Riverside Riverside California USA

**Keywords:** Arbuscular mycorrhizal fungi, climate change, endemic, microhabitat, plant–microbe interactions, population persistence, range shifts, serpentine soils, Siskiyou Mountains, topography

## Abstract

Here we report on a long‐term transplant study of a serpentine endemic plant where individuals were transplanted into cooler macro‐ and microclimatic refugia (i.e., higher elevations and north‐facing aspects) in locations outside of its current range. We describe: (1) how transplanted populations persisted outside of their current range in micro‐ (cooler aspects) or macro‐ (higher elevations) climatic refugia; and (2) soil microbial communities that may have helped or hindered population persistence in climatic refugia. Location: Siskiyou Mountains of southwestern Oregon (USA). Taxon: *Horkelia sericata* (Rosaceae), Angiosperms; Mycota (Fungi); Monera (Bacteria). At each transplant site, we counted surviving individuals (noting reproductive status) and then collected soil from both the rhizosphere of transplanted individuals and from an equal number of areas of nearby bare soil with no plants. Soil bacterial and fungal communities were assessed using next‐generation sequencing of 16S and ITS‐1 marker genes. Of the 15 initial transplant sites, one (high elevation) site displayed population persistence (i.e., “successful” site), defined as having surviving individuals with reproductive success. Four sites had surviving individuals but no reproductive success (i.e., “unsuccessful” sites); the remaining 10 sites had no surviving individuals and were excluded from microbial analyses. The successful site had distinct soil fungal and bacterial community composition (alpha and beta diversity) and a higher mutualist:pathogen ratio than the unsuccessful sites. Additionally, the mutualist:pathogen ratio did not differ between *Horkelia's* rhizosphere and bare ground at the successful site, suggesting that the persistence of this population was potentially enhanced by soil mutualists that were already present at that site. Taken together, these results highlight that the success of species range shifts into climatic refugia may be influenced by the presence of suitable soil microbial communities, with a potentially outsized role of mycorrhizal mutualists, emphasizing the need to consider soil microbial communities in future range predictions of highly specialized plants such as serpentine endemics.

## Graphical abstract

Here we report on a long‐term transplant study of a serpentine endemic plant where individuals were transplanted into areas of climatic refugia in locations outside of its current range. Of the 15 initial transplant sites, one site had surviving individuals with reproductive success and four sites had surviving individuals with no reproductive success after 5 years. Sites with reproductive success (i.e., population persistence) had distinct soil fungal and bacterial community composition and a higher mutualist:pathogen ratio, suggesting that the success of species range shifts into climatic refugia may be influenced by the presence of suitable soil microbes.

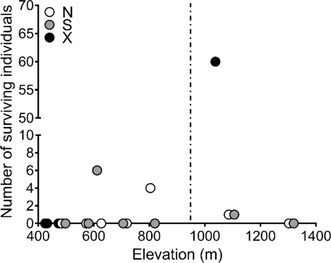

## 1 Introduction

Species are shifting their ranges (i.e., geographic distributions) in response to a changing climate, often moving upward in elevation or latitude to maintain hospitable climate conditions (Parmesan et al. 2003). Range shifts along macroclimatic gradients such as elevation and latitude can allow species to access cooler temperatures and more favorable precipitation regimes (Lenoir and Svenning 2013). Additionally, in heterogeneous landscapes such as montane environments, species may be able to access newly climatically suitable areas over relatively short distances, such as poleward‐facing slopes or valley bottoms (Dobrowski 2011; Ford et al. 2013; Lembrechts 2023). Under current climate change projections, global biodiversity is expected to decline (Urban 2015) and the most at‐risk species have limited dispersal ability (Pearson 2006), restricted ranges (Parmesan 2006), and/or a lower ability to adapt to changing climatic conditions (Davis et al. 2005). Thus, for some species, the ability to move short distances into topographic microrefugia may be critical for their long‐term persistence.

However, even if the distance to a climatically suitable habitat is within reach, other barriers to establishment and persistence in a new location (such as negative biotic interactions and/or an unsuitable soil environment) can limit successful range shifts in response to climate (Sexton et al. 2009; Stanton‐Geddes et al. 2012; Stephan et al. 2021; Fowler et al. 2023). Research has shown that microhabitat can influence seedling establishment during range shifts, and that microhabitat environmental characteristics (e.g., canopy openness, soil nutrients, soil moisture, snow cover) are often decoupled from elevation and/or latitude and macroclimatic patterns (Scherrer and Körner 2010; Chardon et al. 2023). For example, along large climatic and altitudinal gradients in the Cascade Range, WA, USA, only ~33% of the commonly measured components of microhabitat important for plant recruitment followed consistent patterns with elevation (Chardon et al. 2023). Furthermore, even if initial seedling establishment is successful, other biotic and abiotic components of the environment may diminish growth, survival, and reproduction and thus limit population stability and persistence over time (Sheth et al. 2020; McNichol and Russo 2023). Thus, we need an improved understanding of how species range shifts along elevational gradients will interact with changes in biotic and abiotic components of microhabitat.

One component of a microhabitat that is highly influential for establishment and survival, but still poorly understood, is the role of soil microbial communities in inhibiting or facilitating a species' ability to establish and persist in potential refugia. Movement of plants into cooler high‐elevation sites may be mediated by plant–fungi interactions as fungal symbionts (i.e., mutualists) can play an important role in ameliorating harsh abiotic conditions via facilitating water and nutrient uptake (Kivlin et al. 2013; Worchel et al. 2013; Lenoir et al. 2016). Furthermore, as commonly proposed by the “Enemy Release Hypothesis,” plant species may escape from localized soil microbial pathogens during range shifts which may facilitate the establishment of new populations (van Grunsven et al. 2007; Van der Putten et al. 2010; Ramirez et al. 2019). However, by the same token, these species may also leave behind specialized mutualists (i.e., the Missed Mutualist hypothesis) required to establish (Moles et al. 2022).

While predicting the outcomes of species range shifts is rife with uncertainty, small‐scale relocation efforts in topographically complex landscapes have the potential to provide valuable insights into novel biotic interactions outside the home range (Tito et al. 2020). In mountainous landscapes, rugged topography creates large differences in climatic conditions and localized soil microbial communities over short distances (Scherrer and Körner 2010; Dobrowski 2011; De Frenne et al. 2019) while still including the entire home range of many herbivores (Damuth 1981; Behmer 2009) and pollinators (Swarts and Dixon 2009; Menz et al. 2011), thus allowing us to consider shifts in biotic interactions between soil microbes and plants while maintaining other biotic interactions.

Furthermore, for species that are edaphic specialists such as serpentine endemics, differential effects of different biotic soil components may be all the more important for population establishment (Igwe and Vannette 2019), as these species can only tolerate a narrow range of abiotic soil conditions and thus are highly restricted in areas of potential climatic refugia. Previous research has shown that serpentine soils have distinct soil microbial (i.e., bacterial) communities from non‐serpentine soils (Koner et al. 2023; Senthil Kumar et al. 2023), but not fungal or mycorrhizal communities (Branco and Ree 2010; Muller and Hilger 2015). Furthermore, evidence is mixed on whether rhizosphere microbes play a critical role in endemic plant adaptation to serpentine soils (Fitzsimons and Miller 2010; Porter et al. 2017; Igwe and Vannette 2019). Nonetheless, it is unknown how microbial communities may facilitate or limit range shifts of endemic plant species *within* serpentine soils. However, research in non‐serpentine systems suggests that soil microbes can provide locally beneficial interactions and enhance plant success in novel precipitation and soil moisture regimes (Lau and Lennon 2012; Giauque et al. 2019; Allsup and Lankau 2019; Ricks and Yannarell 2023).

Here we report the resampling of a long‐term transplant study of a serpentine endemic plant where individuals were transplanted into cooler macro‐ and microclimatic refugia (i.e., higher elevations and north‐facing aspects) in locations outside of its current range in 2012 (see Spasojevic et al. 2014 for additional details). We resampled these populations five years after the initial transplanting to assess if transplanted populations persisted—noting reproductive status (i.e., flowering, clonal offshoots). Furthermore, we collected soil from both the rhizosphere of transplanted individuals and from an equal number of areas of nearby bare soil with no plants and used next‐generation sequencing of ITS‐1 and 16S marker genes to assess soil fungal and bacterial communities, respectively. We describe: (1) how transplanted populations persisted outside of their current range in micro‐ (cooler aspects) or macro‐ (higher elevations) climatic refugia; and (2) soil microbial communities that may have helped or hindered population persistence in climatic refugia.

We predicted that soil microbial communities would differ in composition (alpha and beta diversity) between home (low elevation, south‐facing) and transplant (high elevation, north‐facing) sites, as well as between the rhizosphere of transplanted individuals and bare soil, reflecting locally adapted soil microbes. We also predicted that mutualist:pathogen ratios would be higher in successful (sites with population persistence‐ i.e., surviving individuals with reproductive success) versus unsuccessful sites (i.e., sites with surviving individuals but no reproductive success) in line with the Enemy Release Hypothesis (van Grunsven et al. 2007; Van der Putten et al. 2010; Ramirez et al. 2019). Finally, we predicted that community composition and the abundance of microbial phyla would differ in concurrence with the Enemy Release Hypothesis, and that the benefits of lower pathogens on population persistence would override the costs of missed mutualists as plant–mutualist relationships are often more generalist than plant–pathogen relationships (Semchenko et al. 2022).

## 2 Materials and Methods

### 2.1 Study System

This study was conducted in the Siskiyou Mountains of southwestern Oregon (USA), an area known for high biodiversity and endemism arising from variation in soils, altitude, topographic relief, and biogeographic history (Whittaker 1960; Damschen et al. 2010). Our sites were located near Cave Junction, Oregon, USA, which has a mean annual temperature of 13.3°C and mean annual precipitation of 160 cm (http://www.wrcc.dri.edu/cgi‐bin/cliMAIN.pl?or1448). This region has a Mediterranean climate and mean annual and seasonal temperatures in this region have increased by 2°C over the past 60 years, total precipitation has not significantly changed, and snowpack has declined (Damschen et al. 2010; Harrison et al. 2010). All of our sites were found on serpentine soils—those that develop on rocks such as peridotite and serpentinite, with high magnesium and iron content (Alexander et al. 2007).

### 2.2 Initial Experimental Design

In 2012, we planted seeds of *Horkelia sericata* (S. Watson—Roseaceae) a rhizomatous perennial plant restricted to serpentine soils at 18 sites along an elevational transect spanning 432–1320 m (Figure S1; see Spasojevic et al. 2014 for details). The original study also included *Arabis aculeolata* (Brassicaceae) and *Phacelia corymbosa* (Boraginaceae), but no individuals of those species survived the resurvey described in this paper (see Section 2.4). Of these sites, 14 were paired north‐ and south‐facing aspects spanning the elevation gradient; one site was a high‐elevation non‐aspect (flat) site, and three were low‐elevation non‐aspect “home” sites where seeds were collected. At each of the 18 sites, we set up 10 plots consisting of two 0.25 m^2^ subplots (*n* = 179 plots total, one plot at one site was lost). Within each subplot, we planted seeds of the target species below the soil surface at four locations in a 4 × 4 grid marked with colored paperclips. Most plots lacked our target species, but at the few home sites where target species was present, we counted only those individuals that germinated within 2 cm of a paperclip. We assessed aboveground biotic interactions in our initial experiment using removal treatments (Spasojevic et al. 2014). Each subplot was randomly assigned to control or neighbor+litter removal. In the neighbor + litter removal subplots, all live and dead aboveground biomass was removed in 2011, prior to the experiment to remove the impact of biotic interactions via live or dead biomass on populations success, and removal was regularly maintained in 2012 and 2013. The removal treatments were not continued past 2013 and the removal and non‐removal plots were indistinguishable in 2017.

### 2.3 Soil Abiotic Conditions

As described in Spasojevic et al. (2014), soils at all sites were dominated by massive‐scaly serpentinite based on thin‐section microscopy and X‐ray diffraction. Elevation and aspect both affected mean and maximum annual soil‐surface temperatures across sites, with ~7°C greater on south‐facing aspects than on north‐facing aspects, equivalent to a ~1000 m elevation gain (Spasojevic et al. 2014). Mean and maximum annual soil‐surface temperatures also declined with increasing elevation; however, there was no interaction between elevation and aspect on soil‐surface temperature. Finally, soil organic matter (OM) content increased with elevation and was higher on north‐facing than south‐facing aspects, but again there was no interaction between elevation and aspect on soil OM content (Spasojevic et al. 2014). In this study, we reanalyzed soil abiotic data for soils collected in 2011 at all sites, including soil pH, Nitrogen (N), Phosphorus (P), Potassium (K), Calcium:Magnesium (Ca:Mg), organic matter content (OM), and cation exchange capacity (CEC), to assess any potential differences between home and away soils, as well as away soils where *Horkelia* populations were successful and unsuccessful.

### 2.4 Plant Resampling

In 2017, we returned to each site and resurveyed each plot for any surviving plants that grew from our transplanted seeds. For sites that initially had none of our target species present, we counted any individual growing at that site within the area of our plots. For sites with target species initially present, we used a conservative estimate and only counted individuals that were in the planting grid and < 2 cm of a colored paperclip that we initially used to mark planted seeds. For any individuals growing in our plots, we noted if they were in flower and/or if they had any clonal offshoots. We assessed a clonal offshoot as small individuals not growing along the grid in which seeds were planted.

### 2.5 Soil Microbial Sampling

In 2017, at all sites with surviving transplanted individuals (i.e., successful and unsuccessful), we collected soils from the rhizosphere of individual plants (*n* = 148) and placed them in sterile Whirlpak bags (Uline, Pleasant Prairie, WI, USA)—sample size varied among sites depending on the number of surviving individuals. We also sampled soils from bare ground at the same site but not under Horkelia individuals. Soils were sampled using a soil corer or hand trowel (depending on the rockiness of the soil) to 10 cm depth, which was sterilized between each sample with a 10% bleach solution to prevent cross‐contamination. At each sampling location, three replicate samples were combined into one sample and all excess rocks, roots, leaves, or twigs were removed. Soils were frozen within 24 h of sampling and remained in the freezer (−20°C) until sequencing prep. We also collected soils from the home site under individual plants as well as bare ground.

### 2.6 Soil Microbial Analysis

Total DNA from soil was extracted from approximately 0.25 g per sample using MoBio PowerSoil DNA extraction kits (MO BIO Laboratories Inc., Carlsbad, CA, USA). Genomic DNA was amplified using the ITS1‐F and ITS2 primers for fungi (McGuire et al. 2013) and the 515‐F and 806‐R primer pair for the 16S rRNA gene in bacteria (Caporaso et al. 2010). PCR was conducted in 25 mL reactions containing 10 mL H_2_O, 12.5 mL GoTAQ 2× MM mix (Promega, Madison, WI, United States), 0.5 mL of both the forward and reverse Illumina barcoded primers, and 1 mL of genomic DNA. PCR cycles were performed at 94°C for 3 min, 35 cycles at 94°C for 45 s, 50°C for 60 s, 72°C for 90 s, then 10 min at 72°C. All the PCR reactions were run in duplicate and visualized using gel electrophoresis. Successful reactions were quantified using a spectrofluorometer, pooled, and sent for Illumina MiSeq paired‐end sequencing.

### 2.7 Bioinformatics

For 16S analyses, raw Illumina reads were de‐multiplexed and processed using QIIME V1.9.1 1 (Caporaso et al. 2010). A total of 61,781,704 paired reads were processed, and all sequences with a quality score < 20 were excluded from the analysis. Operational Taxonomic Units (OTUs) were selected using the UCLUST algorithm and taxonomy was assigned using the Greengenes reference (McDonald et al. 2012) database with the RDP classifier (Wang et al. 2007). The 16S OTU table was rarified to an even depth of 253,827 sequences per sample with the “Phyloseq” package in R (McMurdie and Holmes 2013).

For ITS analyses, raw Illumina reads were demultiplexed and processed using AMPtk V1.4.3 (Palmer et al. 2018). Demultiplexed paired‐end sequences data were pre‐processed by trimming primer sequences, trimming forward and reverse reads to 250 bp (reads length less than 100 bp were dropped), and merging paired‐end reads using USEARCH v.9.2.64 (Edgar 2010).

A total of 9,310,871 reads passed the preprocessing steps, and reads were filtered based on quality scores with a cutoff of an expected error of less than 0.9 (Edgar and Flyvbjerg 2015) to produce 7,304,411 reads which passed quality filtering. The quality‐filtered reads were clustered into 9505 OTUs using UPARSE (Edgar 2013) at a 97% identity threshold. The OTUs were further processed with VSEARCH V2.13.6 (Rognes et al. 2016) to identify and remove 792 chimeras based on comparison to the UNITE database v8.2 (Nilsson et al. 2019) leaving 8711 OTUs. We assigned taxonomy with the AMPtk “hybrid” approach, which uses Global Alignment, SINTAX, and UTAX, and assigned functional guild information to 3405 OTUs using the FUNGuild database (Nguyen et al. 2016). The ITS OTU table was rarified to an even depth of 22,822 sequences per sample with the “Phyloseq” package in R (McMurdie and Holmes 2013).

### 2.8 Statistical Analyses

To test our predictions that soil microbial communities would differ in composition between home and transplant sites, as well as between the rhizosphere of transplanted individuals and bare soil, analyses of soil fungal and bacterial communities were performed in the *phyloseq* package in R (McMurdie and Holmes 2013). First, we tested for differences in alpha diversity and community composition (beta diversity) between sites (home vs. transplant), success (population persistence or not) and source (*Horkelia* rhizosphere vs. bare ground), and their interactions (site × source) and (success × source). For alpha diversity, we used a 2‐way ANOVA to test for differences in mutualist to pathogen ratios between successful and unsuccessful transplant sites, soil sources (*Horkelia* rhizosphere vs. bare ground) and their interaction followed by Tukey's honest significant differences (HSD) tests for pairwise comparisons. For beta diversity, we used a permutational multivariate analysis of variance (perMANOVA) for beta diversity in the vegan function “adonis” in R (999 permutations) (Oksanen et al. 2016) based on Bray–Curtis dissimilarity. Next, to test our prediction that mutualist:pathogen ratios would be higher in successful versus unsuccessful sites, we calculated fungal mutualist to pathogen ratios (both read abundance and richness based) in each soil sample using the FUNGuild assignments (Nguyen et al. 2016) of “Plant Pathogen” for pathogens and “Arbuscular mycorrhizal fungi” (AMF) for mutualists—the primary fungal mutualists of *Horkelia*. We transformed mutualist to pathogen ratios to achieve normality using Tukey's Ladder of Powers in the “tranformTukey” function of the *rcompanion* package in R (Mangiafico 2016). We then used a 2‐way ANOVA to test for differences in mutualist to pathogen ratios between successful and unsuccessful transplant sites, soil sources (*Horkelia* rhizosphere vs. bare ground) and their interaction followed by Tukey's HSD tests for pairwise comparisons.

To test our prediction that the abundance of major microbial phyla would differ between successful and unsuccessful sites, we aggregated the relative read abundances of the top eight bacterial phyla and the top five fungal phyla in all soil samples in the “microbiome” package in R (Lahti and Sudarshan 2017). We log‐transformed and then tested read abundance data for normality at the phylum level using a Shapiro–Wilks test. Bacterial read abundance data were normally distributed, so we used a 2‐way ANOVA test for differences in the relative read abundances for each bacterial phyla (Acidobacteria, Actinobacteria, Planctomycetes, Proteobacteria, and Verrucomicrobia) between successful and unsuccessful transplant sites, soil sources (*Horkelia* rhizosphere vs. bare ground), and their interaction followed by Tukey's HSD tests for pairwise comparisons. For fungal read abundance data which were not normally distributed, we tested for differences in the relative read abundances for each fungal phyla (Ascomycota, Basidiomycota, Glomeromycota, Mucoromycota, and Mortierellomycota) using a Kruskal–Wallace test with the same predictors as above, followed by a Nemenyi test for pairwise comparisons.

Finally, to test for any potential differences in soil abiotic data between sites that may have contributed to differences in *Horkelia* population persistence, we ran one‐way ANOVAs with soil abiotic variables as the dependent variables and soil site (away unsuccessful, away successful, or home) as the independent variable. Response variables were log‐transformed to improve normality. We also report estimates from post hoc testing for Tukey's HSD between the successful and unsuccessful sites. All analyses were run in R statistical software v 4.3.1.

## 3 Results

### 3.1 Plant Population Persistence

**FIGURE 1:**
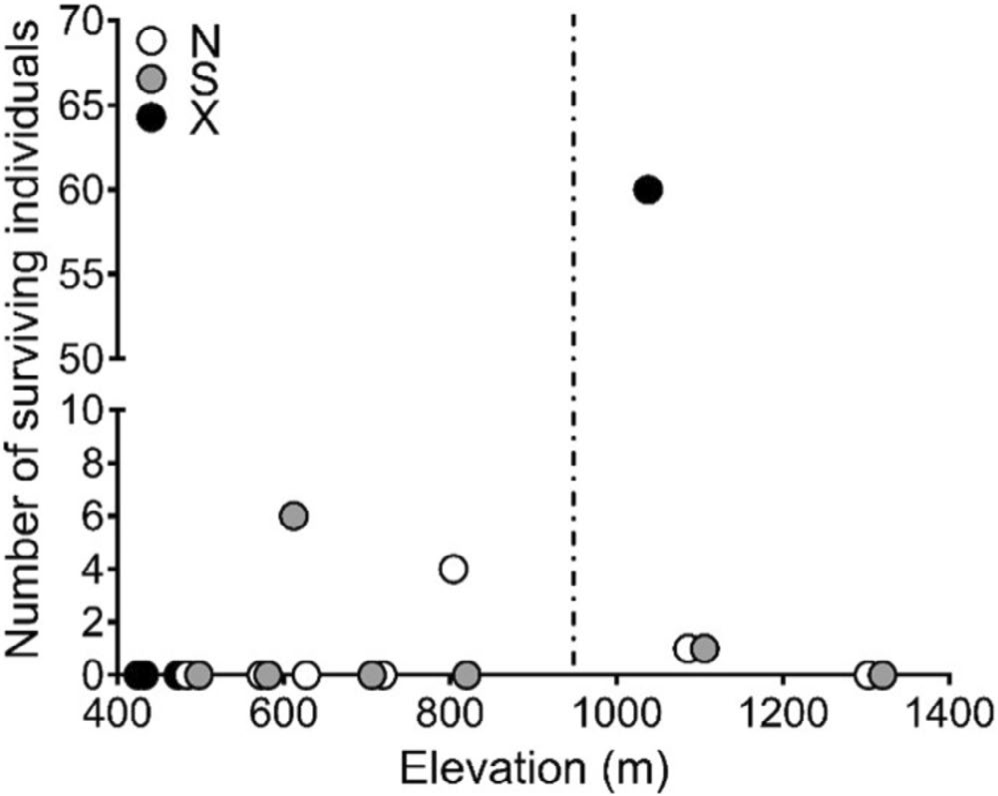
Number of surviving individuals of *Horkelia sericata* (Rosaceae) at each site along an elevation gradient with paired north‐ and south‐facing aspects. Vertical dashed line represents the published upper elevation limit for the *Horkelia*; north‐facing aspects (N: White symbols) south‐facing aspect (S: Gray symbols) and non‐aspect (flat) sites (X: Black symbols). The three low‐elevation flat sites are the home sites where seeds were collected.

We found no surviving individuals of *H. sericata* at most sites, and a very few surviving individuals at two sites on north‐facing aspects (four survivors and one survivor, respectively) and at two sites on south‐facing aspects (six survivors and one survivor, respectively) (Figure 1)—we refer to these as *unsuccessful sites* throughout the rest of the paper. Interestingly, the one higher elevation flat (non‐aspect) site above *Horkelia's* current elevational range had 60 surviving individuals that had grown from seed (four of which were flowering), and 83 clonal offshoots from 33 of those surviving individuals–we refer to this as the *successful site* throughout the rest of the paper. Microclimatic refugia (i.e., north‐facing slopes) did not play a role in population persistence as the successful site was a non‐aspect flat site, and there were an equal number of north‐ and south‐facing unsuccessful sites (two each).

### 3.2 Microbial Taxonomic Analysis

The soil fungal community across all soil samples was comprised of Ascomycota making up the largest percentage (57.4%), followed by Basidiomycota (35.2%), Glomeromycota (4.6%), Mucoromycota (1.8%), Mortierellomycota (0.7%), and other (0.3%). The soil bacterial community across all soil samples was comprised of Proteobacteria making up the largest percentage (22.7%), followed by Acidobacteria (18.7%), Verrucomicrobia (13.9%), Actinobacteria (13.7%), Planctomycetes (10.5%), Bacteroidetes (7.7%), Gemmatimonadetes (5.0%), Chloroflexi (4.3%), and other (3.5%). Overall, 10.2% of the total 16S sequences and 3% of the total ITS sequences had no BLAST hit so taxonomy could not be assigned and thus were excluded from further analyses.

#### 3.2.1 Alpha Diversity

**FIGURE 2:**
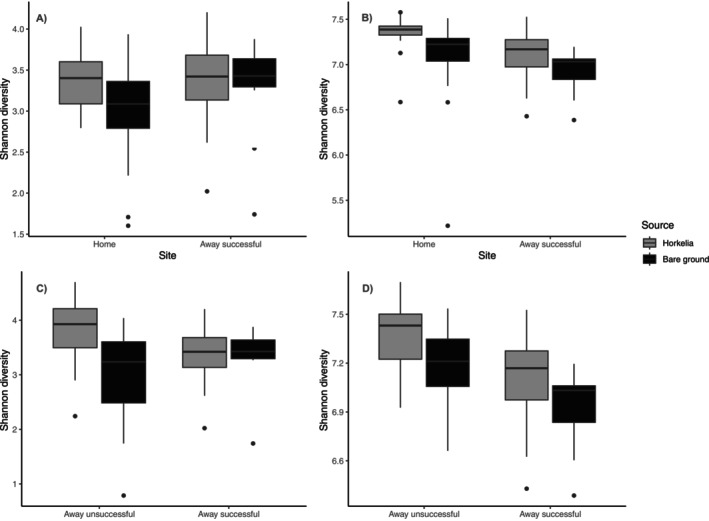
Shannon alpha diversity of fungal (A and C) and bacterial communities (B and D) in sampled soils. Alpha diversity was significantly higher at the home sites and unsuccessful transplant (away) sites than at the successful transplant (away) sites for bacterial communities but did not differ for fungal communities. *Horkelia* soils had higher alpha diversity than bare ground soils overall, but did not differ between *Horkelia* and bare ground at the successful away site only for fungal communities.

We found that the *Horkelia* rhizosphere bacterial communities had higher alpha diversity in the home sites where seeds were collected than the successful transplant site but found no difference for soil fungi between these sites (Bacteria: *F*_1,91_ = 9.124, *p* = 0.003, Figure 2B; Fungi: *F*_1,89_ = 1.311, *p* = 0.254, Figure 2A). Similarly, bacterial alpha diversity was higher in the unsuccessful transplant site than in the successful transplant site (*F*_1,88_ = 26.341, *p* < 0.001, Figure 2D), but there was no difference for soil fungi between these sites (*F*_1,84_ = 0.019, *p* = 0.891, Figure 2C). In terms of soil source, bacterial and fungal alpha diversity was higher in *Horkelia* rhizosphere than bare soils overall (Bacteria: *F*_1,88_ = 13.773, *p* < 0.001, Figure 2B,D; Fungi: *F*_1,84_ = 11.308, *p* = 0.002, Figure 2A,C). However, there was a significant interaction between site (home vs. successful) and soil source for soil fungi but not bacteria, whereby fungal alpha diversity was higher in *Horkelia* rhizosphere than bare soils at the unsuccessful transplant site (*F*_1,84_ = 6.472, *p* = 0.013, Figure 2C, left) and the home site (*F*_1,89_ = 3.493, *p* = 0.063 *marginally significant, Figure 2A, left), but they did not differ between rhizosphere and bare soils at the successful transplant site (Figure 2A,C, right, *p* = 0.99). However, due to limitations with our 16S bioinformatics pipeline, bacterial alpha results should be interpreted with caution (see Section 4.3).

#### 3.2.2 Beta Diversity (Community Composition)

**FIGURE 3:**
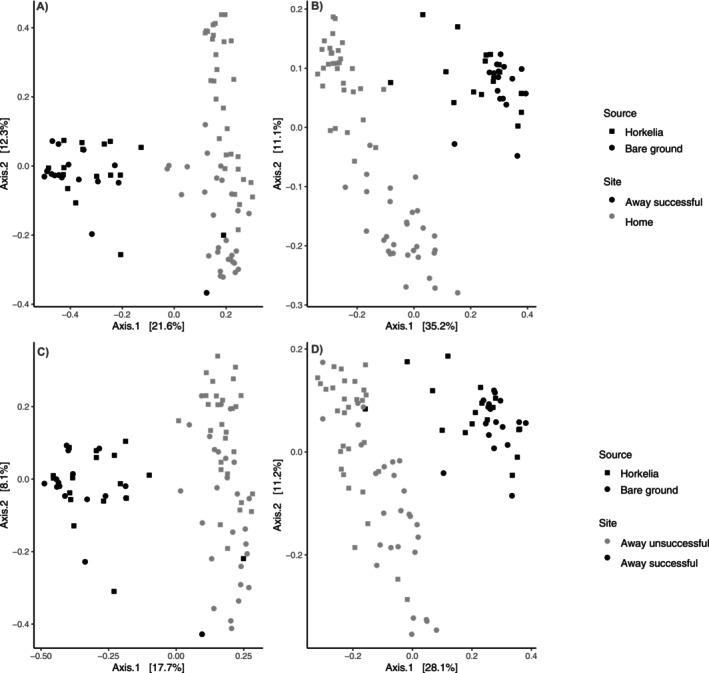
Variation in microbial community composition for fungi (A and C) and bacteria (B and D). Community composition differed between the home sites (gray) where seeds were collected and the successful away site (black) for both fungal (A) and bacterial (B) communities. Moreover, fungal composition (A) differed between Horkelia rhizosphere (squares) and bare soils (circles) but there was no difference between Horkelia rhizosphere and bare soils for bacteria (B). Community composition also differed between the successful away site (black) and the unsuccessful away sites (gray) for both fungal (C) and bacterial (D) composition and fungal composition (C) differed between Horkelia rhizosphere (squares) and bare soils (circles) but there was no difference between Horkelia rhizosphere and bare soils for bacteria (D).

We found that the *Horkelia* rhizosphere bacterial and fungal communities differed between the home sites where seeds were collected and the successful transplant site (Fungi: *F*_1,92_ = 22.57, *p* = 0.001, Figure 3A; Bacteria *F*_1,94_ = 3.33, *p* = 0.007, Figure 3B). Across both home and successful site soils, we found no significant difference between rhizosphere and bare soils for bacteria (*F*_1,94_ = 0.41, *p* = 0.998, Figure 3B), but fungal composition did differ between rhizosphere and bare soils (*F*_1,92_ = 6.74, *p* = 0.001, Figure 3A) and there was a significant interaction between site (home vs. successful) and soil source (rhizosphere vs. bare ground) (*F*_1,92_ = 3.28, *p* = 0.002). However, the differences between rhizosphere and bare ground were driven primarily by the home sites, and when we analyzed fungal composition at just the successful site, we found that fungal composition did not differ between the rhizosphere of *Horkelia* and adjacent bare ground (*F*_1,32_ = 0.64, *p* = 0.92).

Both fungal and bacterial community composition differed between the successful site and the unsuccessful sites (Fungi: *F*_1,87_ = 15.47, *p* = 0.001, Figure 3C; Bacteria *F*_1,91_ = 7.66, *p* = 0.001, Figure 3D). Moreover, across both unsuccessful and successful site soils, we found no significant difference between rhizosphere and bare soils for bacteria (*F*_1,91_ = 0.09, *p* = 0.89, Figure 3D), but fungal composition did differ between rhizosphere and bare soils (*F*_1,87_ = 2.37, *p* = 0.006, Figure 3C).

#### 3.2.3 Functional Guilds

**FIGURE 4:**
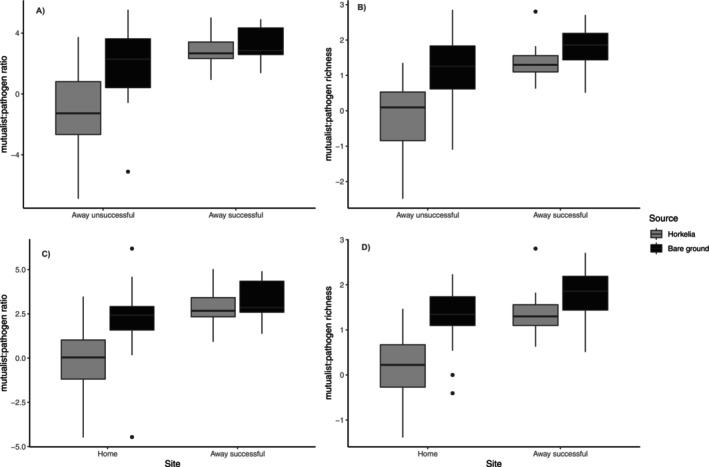
Mutualist to pathogen ratios based on relative read abundance (A and C) and OTU richness (B and D) were significantly higher at the successful away site (right) than at the unsuccessful away sites and home sites (left) but did not differ between *Horkelia* and bare ground at the successful away site.

In total, FUNGuild assigned 267 OTUs as plant mutualists (AMF) and 101 OTUs as plant pathogens, which corresponded to a total of 37 unique mutualistic and 79 unique pathogenic taxa (Table S2). We found that mutualist to pathogen ratios based on abundance (Figure 4A) and richness (Figure 4B) were higher at the successful *Horkelia* sites (*F*_1,84_ = 27.29, *p* < 0.0001; and *F*_1,89_ = 26.49, *p* < 0.0001, respectively) than at unsuccessful sites and higher at the successful *Horkelia* site than at home sites (*F*_1,89_ = 31.57, *p* < 0.0001; *F*_1,89_ = 35.78, *p* < 0.0001, respectively). Mutualist to pathogen ratios based on either abundance or richness did not differ between *Horkelia* and bare ground at the successful site (Tukey Post hoc comparisons, *p* = 0.94 0.35, respectively) or between the unsuccessful away sites and the home sites (Tukey Post hoc comparisons, *p* = 0.13 and 0.19, respectively). The abundance of Glomeromycota (AMF) was higher in successful *Horkelia* sites than non‐successful *Horkelia* sites (KW*χ* ^2^ = 47.87, *p* < 0.001), both overall and in *Horkelia* soils only (*p* < 0.001), but the abundance of Glomeromycota did not differ between *Horkelia* and bare ground at the successful site (*p* = 0.99) (Figure S3, top).

#### 3.2.4 Taxon Relative Abundances

For the remaining microbial phyla, soils from the successful *Horkelia* recipient site had a lower relative abundance of Acidobacteria (*F*_1,91_ = 8.46, *p* = 0.005), but higher Verrucomicrobia (*F*_1,91_ = 22.17, *p* < 0.001) and Planctomycetes (*F*_1,91_ = 87.52, *p* < 0.001) than unsuccessful recipient sites. In addition, *Horkelia* soils at the successful recipient site had a higher abundance of Verrucomicrobia (diff = 0.480, *p* < 0.001) and Planctomycetes (diff = 0.219, *p* < 0.001) than *Horkelia* soils at the unsuccessful recipient sites (Figure S3, bottom). For fungi, soils from the successful *Horkelia* recipient site had higher Ascomycota (KW *χ* ^2^ = 4.33, *p* = 0.037) and Mucoromycota (KW *χ* ^2^ = 25.51, *p* < 0.001) than unsuccessful recipient sites. In addition, *Horkelia* soils at the successful recipient site had a higher abundance of Mucoromycota (Mean rank abundance = 37.09, *p* = 0.009) than *Horkelia* soils at unsuccessful recipient sites (Figure S3, bottom).

### 3.3 Soil Abiotic Conditions

Soil abiotic conditions were relatively similar between the successful and unsuccessful recipient sites. We found no differences in major soil nutrients including N, K, Ca:Mg, and OM content, with the exception of P, which was lower at the successful than the unsuccessful recipient site. Similarly, pH and CEC were also lower at the successful than at the unsuccessful recipient site (Figure S2, Table S1). Broadly, we can say that the successful recipient site was more acidic with lower CEC and P content, reflecting a poorer overall soil quality. Unsurprisingly, we also found differences between home soils and recipient sites, with home soils having a higher pH than successful site soils, lower soil P than unsuccessful site soils, a higher CEC, and lower CA:MG than both recipient sites. Differences in soil abiotic conditions between home and away (recipient) sites are likely due to the strong elevational differences between these sites.

## 4 Discussion

How biotic interactions may facilitate or limit the success of climate‐driven range expansions is a topic of emerging understanding (Littlefield et al. 2019; Stephan et al. 2021; Fowler et al. 2023). Here, we build on an empirical study in a topographically complex landscape which demonstrated that plant establishment in a new range was facilitated by the recipient plant community and that there were no differences in pollinators between home and novel sites (Spasojevic et al. 2014). In addition to these aboveground biotic interactions, we have now explored how belowground plant–microbe interactions may also play a critical role in the population persistence of this range‐shifting plant species. Specifically, we found that microbial communities associated with *H. sericata* (Rosaceae) differed in both alpha and beta diversity between the home sites where seeds were collected and transplant sites, and that our successful site (with population persistence after 5 years) had a higher ratio of mutualists to pathogens than unsuccessful sites. Moreover, the microbial community composition (alpha and beta diversity) and mutualist:pathogen ratio did not differ between the *Horkelia* rhizosphere and bare ground at the successful site. Taken together, these results suggest that soil microbial communities, in particular the presence of key mutualists, may be a key factor in future population success for range‐shifting species.

### 4.1 Population Persistence

We found that only one of the three species that we initially transplanted had populations where individuals survived after 5 years. Since we were unable to conduct annual surveys, we have no data on why relocated individuals of *Arabis* and *Phacelia* did not survive. Despite our prediction that changes in microhabitat on north‐facing slopes would provide climatic microrefugia, we only found a few surviving individuals of *Horkelia* at two sites on north‐facing aspects (four survivors and one survivor, respectively) and at two sites on south‐facing aspects (six survivors and one survivor, respectively). On the other hand, we found 60 surviving individuals at one higher elevation flat (non‐aspect) site. This site initially had no individuals of *Horkelia* when we planted seeds in 2010, is above *Horkelia's* current elevational range, and all individuals were found within the confines of our plots, suggesting that all these individuals had grown from seed that we planted. In the first two years of the experiment, this site had consistently higher overwinter survival and population success than the other sites (Spasojevic et al. 2014), and our longer‐term observations here continue to show population persistence. This persistence was likely due to the unique topography of this site as it was the only high‐elevation site not on a slope. The low water‐holding capacity and OM of serpentine soils (Alexander et al. 2007) typically result in high water flow rates after precipitation events and snowmelt, and the flat topography here may have reduced the impact of either erosion or water flowing downslope away from the target plants. In addition to the 60 surviving individuals of *Horkelia* that we planted, we found that 33 of those 60 individuals had produced between 1 and 3 clonal offshoots each, for a total of 83 additional “individuals” added to this population. Critically, these clonal offshoots have the potential to help buffer this population from extirpation as the predominance of clonal reproduction is one mechanism that has been demonstrated to counteract the extinction of small populations by preserving genetic variation and mitigating the effects of demographic stochasticity (Schaal and Leverich 1996; D'Amato 1997). Moreover, we found that four individuals of *Horkelia* were in flower when we resurveyed the population, suggesting that outcrossing will likely occur in the future. Determining the factors that allow populations of species both to establish *and* persist outside of their current range is critical for making accurate predictions of species distributions under future climates; however many studies (often due to methodological constraints) only assess transplanted populations for less than one generation (Cross and Eckert 2021). Here we provide rare but important information on the success of long(er)‐term persistence of experimental populations beyond their natural range (Cross and Eckert 2021) and highlight the importance of such longer‐term monitoring in future research.

### 4.2 Belowground Interactions

Soil microbes have great potential to influence the success or failure of plant species range shifts through site‐specific differences in mutualists and/or pathogens (Koorem et al. 2020; Rudgers et al. 2020; Mueller et al. 2022). However, there are still many open questions regarding how context‐dependent these processes are when driving changes in plant population and community structure under a rapidly changing climate (Rudgers et al. 2020). Here, we found that the role of soil microbial communities in *Horkelia* population establishment and persistence was highly context‐dependent in that it appeared to facilitate movement into macro‐climatically (higher elevation) cooler sites but not micro‐climatically cooler (north‐facing slopes) sites. Specifically, we found that rhizosphere bacterial and fungal communities differed between the home sites (where seeds were collected) and the successful population site, as well as between the successful and unsuccessful population sites, but at the successful site, the bare ground and the *Horkelia* rhizosphere did not differ. Overall, this suggests that soil microbes at the successful site did not arrive with the *Horkelia* seeds we planted but were already present there. Thus, we can presume that soil microbial communities likely played a role in facilitating the establishment and persistence of *Horkelia* at this higher elevation site.

We also found that our successful site had a higher mutualist to pathogen ratios than at both the home site and the unsuccessful sites in line with our predictions based on the Enemy Release Hypothesis. Both release from pathogen pressure and the presence (or lack) of suitable mutualists may directly impact plant fitness in novel environments (Benning and Moeller 2021). For example, low abundance of symbiotic ectomycorrhizal fungi can inhibit plant colonization (Collier and Bidartondo 2009; Nuñez et al. 2009), but escape from belowground microbial pathogens may facilitate the establishment of new populations (van Grunsven et al. 2007; Van der Putten et al. 2010; Ramirez et al. 2019) while many species may be simultaneously influenced by positive and negative effects of microbial communities (Benning and Moeller 2021). We specifically found that the abundance of Glomeromycota (AMF) was higher in successful *Horkelia* sites than non‐successful *Horkelia* sites, both overall and in *Horkelia* soils only, and that the abundance of Glomeromycota did not differ between *Horkelia* and bare ground at the successful site, indicating the presence of beneficial AMF prior to *Horkelia* establishment. Moreover, many invasive plant species co‐invade novel habitats with their mycorrhizal partners (Nuñez and Dickie 2014); however this is much more common for Ectomycorrhizal and Nitrogen‐fixing species than for Arbuscular Mycorrhizal species such as *H. sericata* . In line with this, post hoc comparisons revealed that mutualist to pathogen ratios based on either abundance or richness did not differ between *Horkelia* and bare ground at the successful site. Together these patterns suggest that the increased success of *Horkelia* at our successful site was correlated with increased mutualism in the soils, and not necessarily decreased pathogens, and that the beneficial mutualists were present prior to *Horkelia* establishment. Indeed, recent reviews have highlighted the critical role that mutualisms can play in shaping species range limits (Fowler et al. 2023; Stephan et al. 2021). Our work provides further insight that the presence of appropriate microbial symbionts in the novel range may be key to long‐term population viability during climate‐driven range shifts.

In addition to fungal mutualists, we found differences in other fungal taxa and bacterial taxa, including a higher abundance of *Verrucomycrobia* and *Planctomycetes*, at the successful transplant site than at the unsuccessful transplant site. These microbial phyla have been shown to have beneficial characteristics for plant growth including carbohydrate metabolism and nitrogen cycling (*Verrucomicrobia*; Bünger et al. 2020) and production of secondary metabolites such as antimicrobial and antinematode compounds that can provide plant protection in the rhizosphere (*Planctomycetes;* Ivanova et al. 2017). Recent work has also shown that these bacterial phyla comprise the top members of the microbial community in serpentine rhizosphere soils (Senthil Kumar et al. 2023) and may play a role in heavy metal tolerance. Thus, the higher abundance of these microbial taxa may have contributed to the population persistence of *H. sericata* at the successful transplant site.

However, while many of the soil abiotic conditions we measured (soil nutrients, OM content) were similar between successful and unsuccessful sites, some variables including soil P, pH, and CEC were different, and thus we cannot fully exclude that these or other unmeasured abiotic soil conditions may have played a role in *Horkelia* establishment and population persistence. Nonetheless, we find it unlikely that this was the primary driver, as the conditions that did differ indicated an overall *poorer* soil quality at the successful site (lower soil P, lower pH, higher CEC), compared to the unsuccessful sites. Thus, we presume that the biotic soil conditions, including in particular higher fungal mutualist abundance and richness, are more likely to have influenced population success at this site. However, in a previous study in this system (Spasojevic et al. 2014), population establishment was strongly influenced by abiotic soil conditions, suggesting that population establishment and persistence may be driven by different aspects of the soil ecosystem; however, only abiotic soil conditions were measured in this previous study. Nonetheless, it is likely that there are distinct limiting factors for population establishment versus persistence and we must consider both when predicting the potential outcomes of climate‐driven range shifts.

### 4.3 Limitations

This descriptive study is hampered by several challenges associated with long‐term studies. First and foremost, survival in nature does not follow a balanced experimental design. We only have one site with true population success (i.e., reproductive individuals) making statistical inference challenging. Second, the bacterial (16S) sequencing data presented in this paper use an older bioinformatics pipeline (QIIME1) that has since been superseded by the improved QIIME 2 platform. While QIIME 1 was a state‐of‐the‐art tool for many years, it has limitations in its OTU clustering algorithm, which can lead to inflated alpha diversity metrics, among others. Thus, the reported descriptions of the bacterial communities in our study system (in particular bacterial alpha diversity) should be interpreted with caution. Finally, the assignment of functional guilds to taxonomic sequencing data is a challenging process for many reasons, including the lack of knowledge of the functional role of many taxa and that many microbial taxa play multiple functional roles in an ecosystem. Thus, applying a single functional guild to a single fungal taxon, as we do with the FUNGuild assignments in this study, is an imperfect approach and certainly underrepresents the full functional complexity of this system.

## 5 Conclusions

This study presents a unique longer‐term view of the potential role of soil microbial communities in facilitating population establishment and persistence of an endemic plant species outside of its current elevational range. We acknowledge that the limited population persistence (one site) limits our ability to generalize beyond that site and limits our statistical power. However, these results provide a valuable, real‐world look into the complexities of species range shifts under climate change, furthering our efforts to move from pattern to process. We find partial support for the Enemy Release Hypothesis as a predictor of native species range shifts, which has received mixed empirical support over the last decade (Mlynarek et al. 2017). However, here we emphasize the beneficial role of enhanced mutualists in certain microhabitats within the larger climatically suitable novel range, rather than the general lack of soil pathogens in the novel range as a whole. Thus, certain microhabitats with beneficial soil microbial communities that differ from the home range may allow for population persistence into areas of climatic refugia. This is an important distinction, as it highlights the role of soil microbial components in microhabitat, a critical biotic filter often not considered in future range predictions (Wisz et al. 2013). Our results also suggest that these critical soil mutualists were already present at the recipient site prior to *Horkelia* establishment, and not brought via the seeds of the transplanted species. Furthermore, we found no indications that soil abiotic conditions were providing beneficial microhabitat at our successful transplant site; if anything, the reverse pattern was true, and beneficial soil microbial communities likely aided plants in acclimating to the harsher soil environment. In conclusion, this work further emphasizes the need to consider biotic interactions as well as microhabitat conditions in shaping species range dynamics, particularly for edaphic specialists whose potential areas of habitat suitability may be significantly reduced under novel climates.

## Author Contributions

**Courtney Collins:** conceptualization (supporting), formal analysis (lead), project administration (equal), visualization (lead), writing – original draft (lead), writing – review and editing (equal). **Devin Dinwiddie:** data curation (equal), formal analysis (equal), resources (equal), software (equal), writing – review and editing (equal). **Nuttapon Pombubpa:** data curation (equal), formal analysis (equal), resources (equal), software (equal), writing – review and editing (equal). **Krista McGuire:** data curation (equal), formal analysis (equal), project administration (supporting), resources (equal), software (equal), supervision (supporting), writing – review and editing (equal). **Marko J. Spasojevic:** conceptualization (lead), data curation (equal), funding acquisition (lead), investigation (lead), methodology (lead), project administration (equal), resources (equal), supervision (equal), visualization (supporting), writing – original draft (supporting), writing – review and editing (equal).

## Conflicts of Interest

The authors declare no conflicts of interest.

## Supporting information


Appendix S1.

## Data Availability

Raw sequencing data are available through the NCBI Short Read Archive Bioproject number: PRJNA1233946. All other data and analysis scripts can be found at the Github repository https://github.com/cour10eygrace/Siskiyou_Microbes or on Zenodo https://doi.org/10.5281/zenodo.15700001.

## References

[ece371629-bib-0001] Alexander, E. B. , R. G. Coleman , T. Keeler‐Wolfe , and S. P. Harrison . 2007. Serpentine Geoecology of Western North America: Geology, Soils, and Vegetation. Oxford University Press.

[ece371629-bib-0002] Allsup, C. , and R. Lankau . 2019. “Migration of Soil Microbes May Promote Tree Seedling Tolerance to Drying Conditions.” Ecology 100, no. 9: e02729. 30991447 10.1002/ecy.2729

[ece371629-bib-0003] Behmer, S. T. 2009. “Insect Herbivore Nutrient Regulation.” Annual Review of Entomology 54: 165–187. 10.1146/annurev.ento.54.110807.090537 18764740

[ece371629-bib-0004] Benning, J. W. , and D. A. Moeller . 2021. “Microbes, Mutualism, and Range Margins: Testing the Fitness Consequences of Soil Microbial Communities Across and Beyond a Native Plant's Range.” New Phytologist 229: 2886–2900. 33225448 10.1111/nph.17102

[ece371629-bib-0005] Branco, S. , and R. H. Ree . 2010. “Serpentine Soils Do Not Limit Mycorrhizal Fungal Diversity.” PLoS One 5: e11757. 20668696 10.1371/journal.pone.0011757 PMC2909254

[ece371629-bib-0006] Bünger, W. , X. Jiang , J. Müller , T. Hurek , and B. Reinhold‐Hurek . 2020. “Novel Cultivated Endophytic Verrucomicrobia Reveal Deep‐Rooting Traits of Bacteria to Associate With Plants.” Scientific Reports 10: 8692. 32457320 10.1038/s41598-020-65277-6 PMC7251102

[ece371629-bib-0007] Caporaso, J. G. , J. Kuczynski , J. Stombaugh , et al. 2010. “Correspondence QIIME Allows Analysis of High‐ Throughput Community Sequencing Data Intensity Normalization Improves Color Calling in SOLiD Sequencing.” Nature Methods 7: 335–336. 20383131 10.1038/nmeth.f.303 PMC3156573

[ece371629-bib-0008] Chardon, N. , L. McBurnie , K. Goodwin , K. Pradhan , J. H. R. Lambers , and A. L. Angert . 2023. “Variable Species Establishment in Response to Microhabitat Indicates Different Likelihoods of Climate‐Driven Range Shifts.” Preprints. 10.22541/au.169685124.46623232/v1.

[ece371629-bib-0009] Collier, F. A. , and M. I. Bidartondo . 2009. “Waiting for Fungi: The Ectomycorrhizal Invasion of Lowland Heathlands.” Journal of Ecology 97: 950–963.

[ece371629-bib-0010] Cross, R. L. , and C. G. Eckert . 2021. “Long‐Term Persistence of Experimental Populations Beyond a Species' Natural Range.” Ecology 102: e03432. 34105785 10.1002/ecy.3432

[ece371629-bib-0011] D'Amato, F. 1997. “Role of Somatic Mutations in the Evolution of Higher Plants.” Caryologia 50: 1–15.

[ece371629-bib-0012] Damschen, E. I. , S. Harrison , and J. B. Grace . 2010. “Climate Change Effects on an Endemic‐Rich Edaphic Flora: Resurveying Robert H. Whittaker's Siskiyou Sites (Oregon, USA).” Ecology 91: 3609–3619. 21302832 10.1890/09-1057.1

[ece371629-bib-0013] Damuth, J. 1981. “Home Range, Home Range Overlap, and Species Energy Use Among Herbivorous Mammals.” Biological Journal of the Linnean Society 15: 185–193.

[ece371629-bib-0014] Davis, M. B. , R. G. Shaw , and J. R. Etterson . 2005. “Evolutionary Responses to Changing Climate.” Ecology 86: 1704–1714.

[ece371629-bib-0015] De Frenne, P. , F. Zellweger , F. Rodríguez‐Sánchez , et al. 2019. “Global Buffering of Temperatures Under Forest Canopies.” Nature Ecology & Evolution 3: 744–749. 30936433 10.1038/s41559-019-0842-1

[ece371629-bib-0016] Dobrowski, S. Z. 2011. “A Climatic Basis for Microrefugia: The Influence of Terrain on Climate.” Global Change Biology 17: 1022–1035.

[ece371629-bib-0017] Edgar, R. C. 2010. “Search and Clustering Orders of Magnitude Faster Than BLAST.” Bioinformatics 26: 2460–2461. 20709691 10.1093/bioinformatics/btq461

[ece371629-bib-0018] Edgar, R. C. 2013. “UPARSE: Highly Accurate OTU Sequences From Microbial Amplicon Reads.” Nature Methods 10: 996–998. 23955772 10.1038/nmeth.2604

[ece371629-bib-0019] Edgar, R. C. , and H. Flyvbjerg . 2015. “Error Filtering, Pair Assembly and Error Correction for Next‐Generation Sequencing Reads.” Bioinformatics 31: 3476–3482. 26139637 10.1093/bioinformatics/btv401

[ece371629-bib-0020] Fitzsimons, M. S. , and R. M. Miller . 2010. “Serpentine Soil has Little Influence on the Root‐Associated Microbial Community Composition of the Serpentine Tolerant Grass Species Avenula Sulcata.” Plant and Soil 330: 393–405.

[ece371629-bib-0021] Ford, K. R. , A. K. Ettinger , J. D. Lundquist , M. S. Raleigh , and J. H. R. Lambers . 2013. “Spatial Heterogeneity in Ecologically Important Climate Variables at Coarse and Fine Scales in a High‐Snow Mountain Landscape.” PLoS One 8: e65008. 23762277 10.1371/journal.pone.0065008 PMC3676384

[ece371629-bib-0022] Fowler, J. C. , M. L. Donald , J. L. Bronstein , and T. E. X. Miller . 2023. “The Geographic Footprint of Mutualism: How Mutualists Influence Species' Range Limits.” Ecological Monographs 93: e1558.

[ece371629-bib-0023] Giauque, H. , E. W. Connor , and C. V. Hawkes . 2019. “Endophyte Traits Relevant to Stress Tolerance, Resource Use and Habitat of Origin Predict Effects on Host Plants.” New Phytologist 221, no. 4: 2239–2249. 30276818 10.1111/nph.15504

[ece371629-bib-0024] Harrison, S. , E. I. Damschen , and J. B. Grace . 2010. “Ecological Contingency in the Effects of Climatic Warming on Forest Herb Communities.” Proceedings of the National Academy of Sciences 107: 19362–19367. 10.1073/pnas.1006823107 PMC2984146 20974978

[ece371629-bib-0025] Igwe, A. N. , and R. L. Vannette . 2019. “Bacterial Communities Differ Between Plant Species and Soil Type, and Differentially Influence Seedling Establishment on Serpentine Soils.” Plant and Soil 441: 423–437.

[ece371629-bib-0077] Ivanova, A. A. , D. G. Naumoff , K. K. Miroshnikov , et al. 2017. “Comparative Genomics of Four Isosphaeraceae Planctomycetes: A Common Pool of Plasmids and Glycoside Hydrolase Genes Shared By Paludisphaera Borealis PX4T, Isosphaera Pallida IS1BT, Singulisphaera Acidiphila DSM 18658t, and Strain SH‐PL62.” Frontiers in Microbiology 8: 412. 28360896 10.3389/fmicb.2017.00412 PMC5352709

[ece371629-bib-0026] Kivlin, S. N. , S. M. Emery , and J. a. Rudgers . 2013. “Fungal Symbionts Alter Plant Responses to Global Change.” American Journal of Botany 100: 1445–1457. 23757444 10.3732/ajb.1200558

[ece371629-bib-0027] Koner, S. , J.‐S. Chen , J. Rathod , B. Hussain , and B.‐M. Hsu . 2023. “Unravelling the Ultramafic Rock‐Driven Serpentine Soil Formation Leading to the Geo‐Accumulation of Heavy Metals: An Impact on the Resident Microbiome, Biogeochemical Cycling and Acclimatized Eco‐Physiological Profiles.” Environmental Research 216: 114664. 36336091 10.1016/j.envres.2022.114664

[ece371629-bib-0028] Koorem, K. , B. L. Snoek , J. Bloem , et al. 2020. “Community‐Level Interactions Between Plants and Soil Biota During Range Expansion.” Journal of Ecology 108: 1860–1873. 32999508 10.1111/1365-2745.13409 PMC7508040

[ece371629-bib-0029] Lahti, L. , and S. Sudarshan . 2017. “Tools for Microbiome Analysis in R. Version.” http://microbiome.github.com/microbiome.

[ece371629-bib-0030] Lau, J. A. , and J. T. Lennon . 2012. “Rapid Responses of Soil Microorganisms Improve Plant Fitness in Novel Environments.” Proceedings of the National Academy of Sciences 109, no. 35: 14058–14062. 10.1073/pnas.1202319109 PMC3435152 22891306

[ece371629-bib-0031] Lembrechts, J. J. 2023. “Microclimate Alters the Picture.” Nature Climate Change 13: 423–424.

[ece371629-bib-0032] Lenoir, I. , J. Fontaine , and A. Lounès‐Hadj Sahraoui . 2016. “Arbuscular Mycorrhizal Fungal Responses to Abiotic Stresses: A Review.” Phytochemistry 123: 4–15. 26803396 10.1016/j.phytochem.2016.01.002

[ece371629-bib-0033] Lenoir, J. , and J.‐C. Svenning . 2013. “Latitudinal and Elevational Range Shifts Under Contemporary Climate Change.” In Encyclopedia of Biodiversity, edited by S. A. Levin , 2nd ed., 599–611. Academic Press.

[ece371629-bib-0034] Littlefield, C. E. , M. Krosby , J. L. Michalak , and J. J. Lawler . 2019. “Connectivity for Species on the Move: Supporting Climate‐Driven Range Shifts.” Frontiers in Ecology and the Environment 17: 270–278.

[ece371629-bib-0035] Mangiafico, S. 2016. “Summary and Analysis of Extension Program Evaluation in R, Version 1.20.05, Revised 2023.” Rutgers Cooperative Extension, New Brunswick, NJ. rcompanion.org/handbook/.

[ece371629-bib-0036] McDonald, D. , M. N. Price , J. Goodrich , et al. 2012. “An Improved Greengenes Taxonomy With Explicit Ranks for Ecological and Evolutionary Analyses of Bacteria and Archaea.” ISME Journal 6: 610–618. 22134646 10.1038/ismej.2011.139 PMC3280142

[ece371629-bib-0037] McGuire, K. L. , S. G. Payne , M. I. Palmer , et al. 2013. “Digging the New York City Skyline: Soil Fungal Communities in Green Roofs and City Parks.” PLoS One 8: e58020. 23469260 10.1371/journal.pone.0058020 PMC3585938

[ece371629-bib-0038] McMurdie, P. J. , and S. Holmes . 2013. “Phyloseq: An R Package for Reproducible Interactive Analysis and Graphics of Microbiome Census Data.” PLoS One 8: e61217. 23630581 10.1371/journal.pone.0061217 PMC3632530

[ece371629-bib-0039] McNichol, B. H. , and S. E. Russo . 2023. “Plant Species' Capacity for Range Shifts at the Habitat and Geographic Scales: A Trade‐Off‐Based Framework.” Plants 12: 1248. 36986935 10.3390/plants12061248 PMC10056461

[ece371629-bib-0040] Menz, M. H. M. , R. D. Phillips , R. Winfree , et al. 2011. “Reconnecting Plants and Pollinators: Challenges in the Restoration of Pollination Mutualisms.” Trends in Plant Science 16: 4–12. 20980193 10.1016/j.tplants.2010.09.006

[ece371629-bib-0041] Mlynarek, J. J. , C. E. Moffat , S. Edwards , et al. 2017. “Enemy Escape: A General Phenomenon in a Fragmented Literature?” Facets 2: 1015–1044.

[ece371629-bib-0042] Moles, A. T. , R. L. Dalrymple , S. Raghu , S. P. Bonser , and J. Ollerton . 2022. “Advancing the Missed Mutualist Hypothesis, the Under‐Appreciated Twin of the Enemy Release Hypothesis.” Biology Letters 18: 20220220. 36259169 10.1098/rsbl.2022.0220 PMC9579764

[ece371629-bib-0043] Mueller, T. L. , E. Karlsen‐Ayala , D. A. Moeller , and J. Bellemare . 2022. “Of Mutualism and Migration: Will Interactions With Novel Ericoid Mycorrhizal Communities Help or Hinder Northward Rhododendron Range Shifts?” Oecologia 198: 839–852. 34974625 10.1007/s00442-021-05081-9 PMC9056439

[ece371629-bib-0044] Muller, L. A. H. , and H. H. Hilger . 2015. “Insights Into the Effects of Serpentine Soil Conditions on the Community Composition of Fungal Symbionts in the Roots of *Onosma echioides* .” Soil Biology and Biochemistry 81: 1–8.

[ece371629-bib-0045] Nguyen, N. H. , Z. Song , S. T. Bates , et al. 2016. “FUNGuild: An Open Annotation Tool for Parsing Fungal Community Datasets by Ecological Guild.” Fungal Ecology 20: 241–248.

[ece371629-bib-0046] Nilsson, R. H. , K. H. Larsson , A. F. S. Taylor , et al. 2019. “The UNITE Database for Molecular Identification of Fungi: Handling Dark Taxa and Parallel Taxonomic Classifications.” Nucleic Acids Research 47: D259–D264. 30371820 10.1093/nar/gky1022 PMC6324048

[ece371629-bib-0047] Nuñez, M. A. , and I. A. Dickie . 2014. “Invasive Belowground Mutualists of Woody Plants.” Biological Invasions 16: 645–661.

[ece371629-bib-0048] Nuñez, M. a. , T. R. Horton , and D. Simberloff . 2009. “Lack of Belowground Mutualisms Hinders Pinaceae Invasions.” Ecology 90: 2352–2359. 19769113 10.1890/08-2139.1

[ece371629-bib-0049] Oksanen, J. , F. Blanchet , R. Kindt , P. Legendre , and R. O'Hara . 2016. “Vegan: Community Ecology Package. R Package 2.3‐3.” https://cran.r‐project.org/web/packa.

[ece371629-bib-0050] Palmer, J. M. , M. A. Jusino , M. T. Banik , and D. L. Lindner . 2018. “Non‐Biological Synthetic Spike‐In Controls and the AMPtk Software Pipeline Improve Mycobiome Data.” PeerJ 6: e4925. 29868296 10.7717/peerj.4925 PMC5978393

[ece371629-bib-0051] Parmesan, C. 2006. “Ecological and Evolutionary Responses to Recent Climate Change.” Annual Review of Ecology, Evolution, and Systematics 37: 637–669.

[ece371629-bib-0052] Parmesan, C. , C. Parmesan , G. Yohe , and G. Yohe . 2003. “A Globally Coherent Fingerprint of Climate Change Impacts Across Natural Systems.” Nature 421: 37–42. 12511946 10.1038/nature01286

[ece371629-bib-0053] Pearson, R. G. 2006. “Climate Change and the Migration Capacity of Species.” Trends in Ecology & Evolution 21, no. 3: 111–113. 16701483 10.1016/j.tree.2005.11.022

[ece371629-bib-0054] Porter, S. S. , P. L. Chang , C. A. Conow , J. P. Dunham , and M. L. Friesen . 2017. “Association Mapping Reveals Novel Serpentine Adaptation Gene Clusters in a Population of Symbiotic Mesorhizobium.” ISME Journal 11: 248–262. 27420027 10.1038/ismej.2016.88 PMC5315480

[ece371629-bib-0055] Ramirez, K. S. , L. B. Snoek , K. Koorem , et al. 2019. “Range‐Expansion Effects on the Belowground Plant Microbiome.” Nature Ecology & Evolution 3: 604–611. 30911144 10.1038/s41559-019-0828-z PMC6443080

[ece371629-bib-0056] Ricks, K. D. , and A. C. Yannarell . 2023. “Soil Moisture Incidentally Selects for Microbes That Facilitate Locally Adaptive Plant Response.” Proceedings of the Royal Society B: Biological Sciences 290, no. 2001: 20230469. 10.1098/rspb.2023.0469 PMC10291722 37357863

[ece371629-bib-0057] Rognes, T. , T. Flouri , B. Nichols , C. Quince , and F. Mahé . 2016. “VSEARCH: A Versatile Open Source Tool for Metagenomics.” PeerJ 4: e2584. 27781170 10.7717/peerj.2584 PMC5075697

[ece371629-bib-0058] Rudgers, J. A. , M. E. Afkhami , L. Bell‐Dereske , et al. 2020. “Climate Disruption of Plant‐Microbe Interactions.” Annual Review of Ecology, Evolution, and Systematics 51: 561–586.

[ece371629-bib-0059] Schaal, B. A. , and W. J. Leverich . 1996. “Molecular Variation in Isolated Plant Populations.” Plant Species Biology 11: 33–40.

[ece371629-bib-0060] Scherrer, D. , and C. Körner . 2010. “Infra‐Red Thermometry of Alpine Landscapes Challenges Climatic Warming Projections.” Global Change Biology 16: 2602–2613.

[ece371629-bib-0061] Semchenko, M. , K. E. Barry , F. T. de Vries , L. Mommer , M. Moora , and J. G. Maciá‐Vicente . 2022. “Deciphering the Role of Specialist and Generalist Plant–Microbial Interactions as Drivers of Plant–Soil Feedback.” New Phytologist 234: 1929–1944. 35338649 10.1111/nph.18118

[ece371629-bib-0062] Senthil Kumar, R. , S. Koner , H.‐C. Tsai , J.‐S. Chen , S.‐W. Huang , and B.‐M. Hsu . 2023. “Deciphering Endemic Rhizosphere Microbiome Community's Structure Towards the Host‐Derived Heavy Metals Tolerance and Plant Growth Promotion Functions in Serpentine Geo‐Ecosystem.” Journal of Hazardous Materials 452: 131359. 37031672 10.1016/j.jhazmat.2023.131359

[ece371629-bib-0063] Sexton, J. P. , P. J. McIntyre , A. L. Angert , and K. J. Rice . 2009. “Evolution and Ecology of Species Range Limits.” Annual Review of Ecology, Evolution, and Systematics 40: 415–436.

[ece371629-bib-0064] Sheth, S. N. , N. Morueta‐Holme , and A. L. Angert . 2020. “Determinants of Geographic Range Size in Plants.” New Phytologist 226: 650–665. 31901139 10.1111/nph.16406

[ece371629-bib-0065] Spasojevic, M. J. , S. Harrison , H. W. Day , and R. J. Southard . 2014. “Above‐ and Belowground Biotic Interactions Facilitate Relocation of Plants Into Cooler Environments.” Ecology Letters 17: 700–709. 24641109 10.1111/ele.12272

[ece371629-bib-0066] Stanton‐Geddes, J. , P. Tiffin , and R. G. Shaw . 2012. “Role of Climate and Competitors in Limiting Fitness Across Range Edges of an Annual Plant.” Ecology 93: 1604–1613. 22919907 10.1890/11-1701.1

[ece371629-bib-0067] Stephan, P. , B. Bramon Mora , and J. M. Alexander . 2021. “Positive Species Interactions Shape Species' Range Limits.” Oikos 130: 1611–1625.

[ece371629-bib-0068] Swarts, N. D. , and K. W. Dixon . 2009. “Terrestrial Orchid Conservation in the Age of Extinction.” Annals of Botany 104: 543–556. 19218582 10.1093/aob/mcp025 PMC2720663

[ece371629-bib-0069] Tito, R. , H. L. Vasconcelos , and K. J. Feeley . 2020. “Mountain Ecosystems as Natural Laboratories for Climate Change Experiments.” Frontiers in Forests and Global Change 3: 38.

[ece371629-bib-0070] Urban, M. C. 2015. “Accelerating Extinction Risk From Climate Change.” Science 348: 571–573. 25931559 10.1126/science.aaa4984

[ece371629-bib-0071] Van der Putten, W. H. , M. Macel , and M. E. Visser . 2010. “Predicting Species Distribution and Abundance Responses to Climate Change: Why It Is Essential to Include Biotic Interactions Across Trophic Levels.” Philosophical Transactions of the Royal Society, B: Biological Sciences 365: 2025–2034. 10.1098/rstb.2010.0037 PMC2880132 20513711

[ece371629-bib-0072] van Grunsven, R. H. A. , W. H. van der Putten , T. M. Bezemer , W. L. M. Tamis , F. Berendse , and E. M. Veenendaal . 2007. “Reduced Plant‐Soil Feedback of Plant Species Expanding Their Range as Compared to Natives.” Journal of Ecology 95: 1050–1057.

[ece371629-bib-0073] Wang, Q. , G. M. Garrity , J. M. Tiedje , and J. R. Cole . 2007. “Naive Bayesian Classifier for Rapid Assignment of rRNA Sequences Into the New Bacterial Taxonomy.” Applied and Environmental Microbiology 73: 5261–5267. 17586664 10.1128/AEM.00062-07 PMC1950982

[ece371629-bib-0074] Whittaker, R. H. 1960. “Vegetation of the Siskiyou Mountains, Oregon and California.” Ecological Monographs 30: 279–338.

[ece371629-bib-0075] Wisz, M. S. , J. Pottier , W. D. Kissling , et al. 2013. “The Role of Biotic Interactions in Shaping Distributions and Realised Assemblages of Species: Implications for Species Distribution Modelling.” Biological Reviews of the Cambridge Philosophical Society 88: 15–30. 22686347 10.1111/j.1469-185X.2012.00235.x PMC3561684

[ece371629-bib-0076] Worchel, E. R. , H. E. Giauque , and S. N. Kivlin . 2013. “Fungal Symbionts Alter Plant Drought Response.” Microbial Ecology 65: 671–678. 23250115 10.1007/s00248-012-0151-6

