# Supplementary material for: Population Persistence and Soil Microbial Communities of a Serpentine Endemic Plant Outside Its Historic Elevation Range
Source: Ecol Evol. 2025 Jun 21;15(6):e71629. doi: 10.1002/ece3.71629 (PMC12181681; doi:10.1002/ece3.71629)
Supplement: Supplementary file 1 — Appendix S1. [file ECE3-15-e71629-s001.zip › Supplement_revised.docx]

**Figure S1. Transplant experimental design.** Schematic of montane elevation gradient with 15 transplant sites (higher elevation) and 3 home sites (#1, 9, 10- low elevation) respectively. 14 transplant sites were paired on North (N- white circle) and South (S-grey circle) facing aspects and one transplant site (#5- black circle) was on a flat aspect. Seeds of *Horkelia* were collected from home sites (#1, 9, 10- low elevation). Modified from Spasojevic *et al*. 2014.


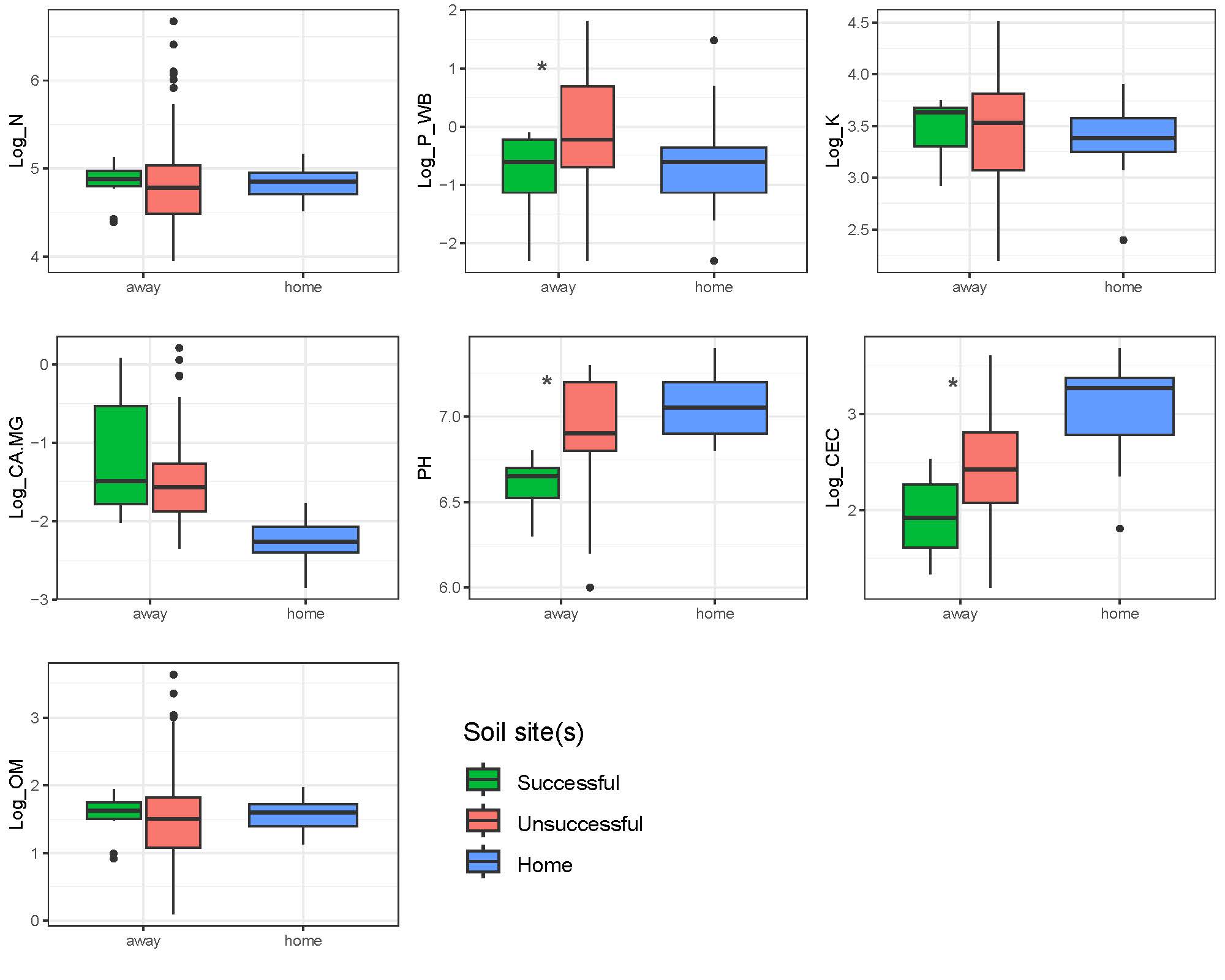


**Figure S2. Soil environmental data.** Box and Whiskers plot of soil environmental (i.e. abiotic) data for the following variables: Nitrogen (N), Phosphorus (P), Potassium (K), Calcium: Magnesium (Ca:Mg), pH, Cation Exchange Capacity (CEC), Organic matter (OM) content. Color indicates which site the soil was sampled at (Away unsuccessful, Away successful, or Home) and asterisks (*) are shown where there was a significant difference between Successful and Unsuccessful soils (see Table S1). Variables are plotted on the log scale.


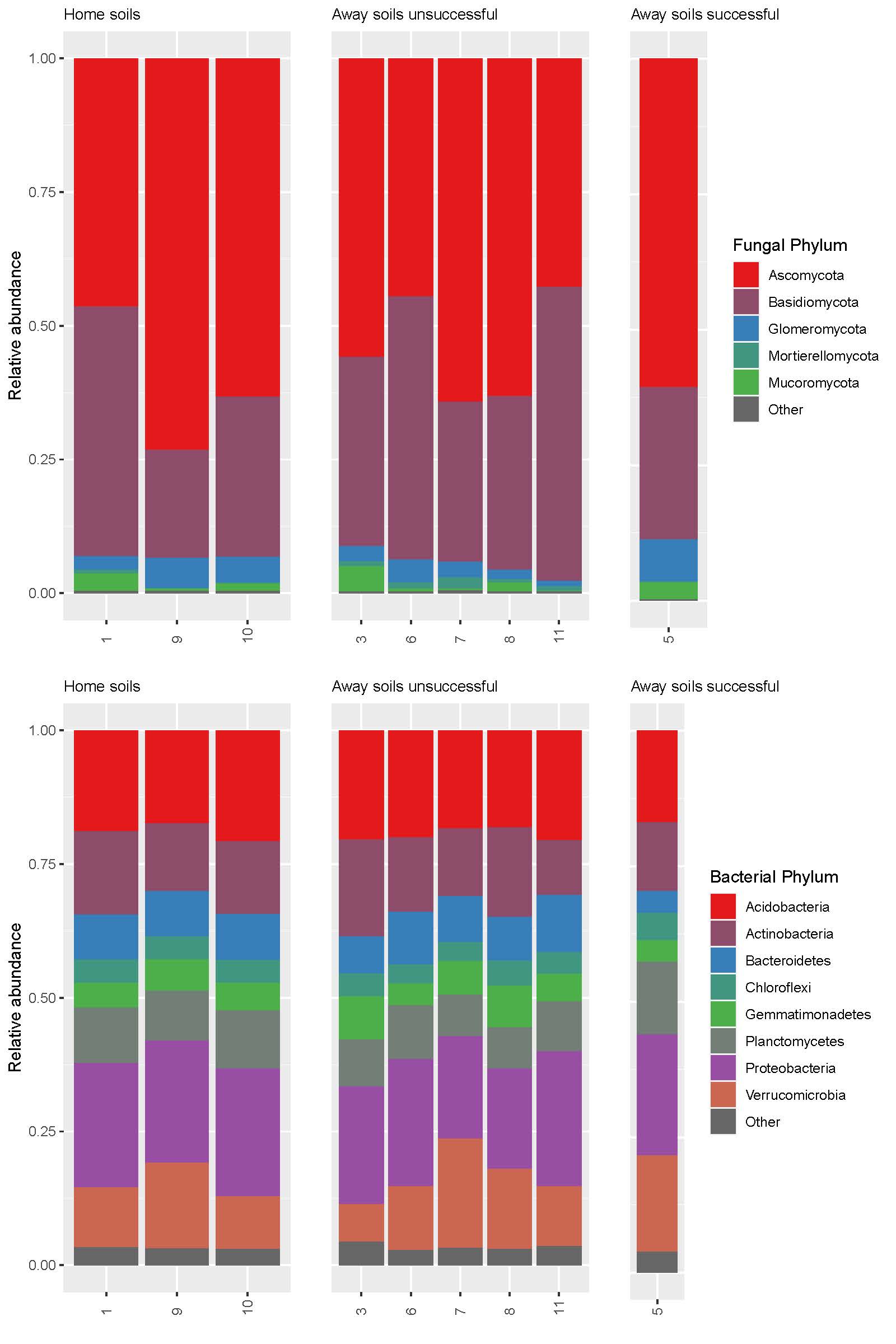


**Figure S3**. Relative abundances of major soil microbial phyla (fungi-top, bacteria-bottom) sampled from the rhizosphere of Horkelia seedlings at each site including the 3 home sites where seeds were collected for transplanting, 5 sites where remnant individuals remained 5 years after transplanting but no population persistence occurred (i.e. ‘unsuccessful’), and the 1 high elevation ‘successful’ site where the Horkelia population persisted 5 years after transplanting. ‘Other’ reflects sequences that were not assigned.

**Table S1.** Results of one-way ANOVAs with soil environmental variables as the dependent variables and soil site (Away unsuccessful, Away successful, or Home) as the independent variable. Response variables were logged to improve normality. We also report estimates from post-hoc testing for Tukey’s honest significant differences (HSD) between the successful and unsuccessful sites. Significance at α < 0.05 is indicated with an asterisk (*).

| Metric | Sum Squared error (SSE) | F value | P value | Successful – Unsuccessful contrast |
| --- | --- | --- | --- | --- |
| Nitrogen (N) | 0.011 | 0.025 | 0.975 | 0.014 (NS) |
| Phosphorus (P) | 10.97 | 6.94 | 0.001* | -0.71* |
| Potassium (K) | 0.12 | 0.294 | 0.746 | 0.99 (NS) |
| Calcium: Magnesium (Ca:Mg) | 15.98 | 31.05 | <0.001* | 0.279 (NS) |
| pH | 1.416 | 10.74 | <0.001* | -0.308 * |
| Cation Exchange Capacity (CEC) | 14.34 | 32.47 | <0.001* | -0.502* |
| Organic matter (OM) content | 0.16 | 0.212 | 0.809 | 0.059 (NS) |

Table S2. FUNGuild taxonomic assignments for a) plant mutualists (Arbuscular Mycorrhizal Fungi) and b) plant pathogens in all soil samples (n=148).

A) Mutualists

| **Phylum** | **Class** | **Order** | **Family** | **Genus** | **Species 1** | **Species 2** |
| --- | --- | --- | --- | --- | --- | --- |
| Glomeromycota | Glomeromycetes | Glomerales | Glomeraceae | Glomus | NA | NA |
| Glomeromycota | Glomeromycetes | Glomerales | Glomeraceae | NA | NA | NA |
| Glomeromycota | Glomeromycetes | Diversisporales | Diversisporaceae | Diversispora | NA | NA |
| Glomeromycota | Glomeromycetes | Diversisporales | Diversisporaceae | NA | NA | NA |
| Glomeromycota | Paraglomeromycetes | Paraglomerales | Paraglomeraceae | Paraglomus | NA | NA |
| Glomeromycota | Glomeromycetes | Glomerales | NA | NA | NA | NA |
| Glomeromycota | Glomeromycetes | Glomerales | Glomeraceae | Rhizophagus | NA | NA |
| Glomeromycota | Glomeromycetes | Glomerales | Glomeraceae | Dominikia | Dominikia | Iranica |
| Glomeromycota | Glomeromycetes | Glomerales | Glomeraceae | Dominikia | Dominikia | difficilevidera |
| Glomeromycota | Archaeosporomycetes | Archaeosporales | NA | NA | NA | NA |
| Glomeromycota | Glomeromycetes | Glomerales | Claroideoglomeraceae | Claroideoglomus | NA | NA |
| Glomeromycota | Glomeromycetes | Diversisporales | Acaulosporaceae | NA | NA | NA |
| Glomeromycota | Glomeromycetes | Gigasporales | Gigasporaceae | Cetraspora | Cetraspora | gilmorei |
| Glomeromycota | Glomeromycetes | Glomerales | Glomeraceae | Rhizophagus | Rhizophagus | irregularis |
| Glomeromycota | Glomeromycetes | Diversisporales | Acaulosporaceae | Acaulospora | NA | NA |
| Glomeromycota | NA | NA | NA | NA | NA | NA |
| Glomeromycota | Glomeromycetes | Gigasporales | Gigasporaceae | NA | NA | NA |
| Glomeromycota | Glomeromycetes | Glomerales | Glomeraceae | Glomus | Glomus | macrocarpum |
| Glomeromycota | Archaeosporomycetes | Archaeosporales | Archaeosporaceae | NA | NA | NA |
| Glomeromycota | Glomeromycetes | Glomerales | Glomeraceae | Rhizophagus | Rhizophagus | intraradices |
| Glomeromycota | Glomeromycetes | Diversisporales | Acaulosporaceae | Acaulospora | Acaulospora | Nivalis |
| Glomeromycota | Glomeromycetes | Glomerales | Claroideoglomeraceae | Claroideoglomus | Claroideoglomus | claroideum |
| Glomeromycota | Glomeromycetes | Glomerales | Glomeraceae | Funneliformis | NA | NA |
| Glomeromycota | Glomeromycetes | Glomerales | Glomeraceae | Glomus | Glomus | indicum |
| Glomeromycota | Glomeromycetes | Diversisporales | Diversisporaceae | Otospora | Otospora | bareae |
| Glomeromycota | Glomeromycetes | Diversisporales | Pacisporaceae | Pacispora | Pacispora | scintillans |
| Glomeromycota | Archaeosporomycetes | Archaeosporales | Archaeosporaceae | Palaeospora | NA | NA |
| Glomeromycota | Glomeromycetes | Gigasporales | Gigasporaceae | Scutellospora | NA | NA |
| Glomeromycota | Glomeromycetes | Glomerales | Glomeraceae | Funneliformis | Funneliformis | mosseae |
| Glomeromycota | Glomeromycetes | Glomerales | Claroideoglomeraceae | Claroideoglomus | Claroideoglomus | lamellosum |
| Glomeromycota | Archaeosporomycetes | Archaeosporales | Ambisporaceae | Ambispora | NA | NA |
| Glomeromycota | Glomeromycetes | Glomerales | Claroideoglomeraceae | NA | NA | NA |
| Glomeromycota | Glomeromycetes | Diversisporales | NA | NA | NA | NA |
| Glomeromycota | Glomeromycetes | Diversisporales | Diversisporaceae | Diversispora | Diversispora | eburnea |
| Glomeromycota | Glomeromycetes | Diversisporales | Acaulosporaceae | Acaulospora | Acaulospora | brasiliensis |
| Glomeromycota | Glomeromycetes | Gigasporales | Gigasporaceae | Cetraspora | NA | NA |
| Glomeromycota | Glomeromycetes | Glomerales | Glomeraceae | Glomus | Glomus | sinuosum |

B) Plant Pathogens

| **Phylum** | **Class** | **Order** | **Family** | **Genus** | **Species 1** | **Species 2** |
| --- | --- | --- | --- | --- | --- | --- |
| Ascomycota | Dothideomycetes | Capnodiales | Mycosphaerellaceae | Mycosphaerella | NA | NA |
| Ascomycota | Sordariomycetes | Xylariales | Bartaliniaceae | Truncatella | Truncatella | Angustata |
| Ascomycota | Sordariomycetes | Hypocreales | Nectriaceae | Volutella | NA | NA |
| Ascomycota | Dothideomycetes | Venturiales | Venturiaceae | Venturia | NA | NA |
| Ascomycota | Dothideomycetes | Capnodiales | Mycosphaerellaceae | Septoria | NA | NA |
| Ascomycota | Dothideomycetes | Pleosporales | Massarinaceae | Stagonospora | Stagonospora | Bicolor |
| Ascomycota | Leotiomycetes | Phacidiales | Phacidiaceae | Phacidium | NA | NA |
| Ascomycota | Taphrinomycetes | Taphrinales | Taphrinaceae | Taphrina | Taphrina | tormentillae |
| Ascomycota | Taphrinomycetes | Taphrinales | Protomycetaceae | Protomyces | Protomyces | inouyei |
| Ascomycota | Dothideomycetes | Botryosphaeriales | Botryosphaeriaceae | Macrophomina | NA | NA |
| Ascomycota | Sordariomycetes | Hypocreales | Bionectriaceae | Clonostachys | NA | NA |
| Ascomycota | Dothideomycetes | Dothideales | Dothideaceae | Scleroconidioma | NA | NA |
| Ascomycota | Leotiomycetes | Helotiales | Sclerotiniaceae | NA | NA | NA |
| Ascomycota | Dothideomycetes | Pleosporales | Didymellaceae | Ascochyta | NA | NA |
| Ascomycota | Leotiomycetes | Helotiales | Sclerotiniaceae | Sclerotinia | NA | NA |
| Ascomycota | Dothideomycetes | Pleosporales | Pleosporaceae | Curvularia | NA | NA |
| Ascomycota | Dothideomycetes | Pleosporales | Pleosporaceae | Pyrenophora | Pyrenophora | seminiperda |
| Ascomycota | Sordariomycetes | Hypocreales | Nectriaceae | Volutella | Volutella | ciliata |
| Ascomycota | Dothideomycetes | Capnodiales | Teratosphaeriaceae | Bryochiton | Bryochiton | perpusillus |
| Ascomycota | Dothideomycetes | Capnodiales | Mycosphaerellaceae | Ramularia | NA | NA |
| Ascomycota | Dothideomycetes | Pleosporales | Didymellaceae | Phoma | Phoma | herbarum |
| Ascomycota | Dothideomycetes | Pleosporales | Pleosporaceae | Drechslera | NA | NA |
| Ascomycota | Dothideomycetes | Botryosphaeriales | Botryosphaeriaceae | Macrophomina | Macrophomina | phaseolina |
| Basidiomycota | Tremellomycetes | Trichosporonales | Tetragoniomycetaceae | Cryptotrichosporon | NA | NA |
| Ascomycota | Dothideomycetes | Capnodiales | Mycosphaerellaceae | Ragnhildiana | NA | NA |
| Ascomycota | Leotiomycetes | Helotiales | Helotiaceae | Collophora | NA | NA |
| Ascomycota | Sordariomycetes | Glomerellales | Plectosphaerellaceae | Gibellulopsis | Gibellulopsis | nigrescens |
| Ascomycota | Sordariomycetes | Magnaporthales | Magnaporthaceae | Slopeiomyces | Slopeiomyces | cylindrosporus |
| Ascomycota | Dothideomycetes | Pleosporales | Pleosporaceae | Curvularia | Curvularia | protuberata |
| Ascomycota | Leotiomycetes | Rhytismatales | Rhytismataceae | Lophodermium | Lophodermium | baculiferum |
| Ascomycota | Sordariomycetes | Xylariales | Amphisphaeriaceae | Seimatosporium | Seimatosporium | vitis |
| Ascomycota | Leotiomycetes | Rhytismatales | Rhytismataceae | Elytroderma | Elytroderma | deformans |
| Ascomycota | Leotiomycetes | Rhytismatales | Rhytismataceae | Lophodermium | NA | NA |
| Ascomycota | Eurotiomycetes | Chaetothyriales | Herpotrichiellaceae | Veronaea | NA | NA |
| Basidiomycota | Agaricomycetes | Hymenochaetales | Rickenellaceae | Rickenella | Rickenella | fibula |
| Ascomycota | Dothideomycetes | Pleosporales | Pleosporaceae | Bipolaris | Bipolaris | simmondsii |
| Basidiomycota | Agaricomycetes | Cantharellales | Ceratobasidiaceae | Thanatephorus | NA | NA |
| Ascomycota | Sordariomycetes | Xylariales | Hyponectriaceae | Monographella | Monographella | nivalis |
| Ascomycota | Sordariomycetes | Diaporthales | Valsaceae | Valsa | Valsa | pini |
| Ascomycota | Sordariomycetes | Glomerellales | Plectosphaerellaceae | Plectosphaerella | Plectosphaerella | cucumerina |
| **Phylum** | **Class** | **Order** | **Family** | **Genus** | **Species 1** | **Species 2** |
| Ascomycota | Eurotiomycetes | Phaeomoniellales | Phaeomoniellaceae | Phaeomoniella | NA | NA |
| Ascomycota | Leotiomycetes | Helotiales | Sclerotiniaceae | Mycopappus | NA | NA |
| Ascomycota | Dothideomycetes | Pleosporales | Didymellaceae | Stagonosporopsis | NA | NA |
| Ascomycota | Sordariomycetes | Xylariales | Bartaliniaceae | Truncatella | NA | NA |
| Ascomycota | Leotiomycetes | Helotiales | Sclerotiniaceae | Pycnopeziza | NA | NA |
| Ascomycota | Dothideomycetes | Venturiales | Venturiaceae | Cylindrosympodium | NA | NA |
| Ascomycota | Dothideomycetes | Capnodiales | Teratosphaeriaceae | Penidiella | Penidiella | ellipsoidea |
| Ascomycota | Sordariomycetes | Xylariales | Amphisphaeriaceae | Monochaetia | Monochaetia | monochaeta |
| Basidiomycota | Agaricomycetes | Agaricales | Chondrostereum | Chondrostereum | NA | NA |
| Ascomycota | Sordariomycetes | Hypocreales | Nectriaceae | Neonectria | Neonectria | major |
| Ascomycota | Sordariomycetes | Hypocreales | Nectriaceae | Neonectria | NA | NA |
| Ascomycota | Dothideomycetes | Capnodiales | Mycosphaerellaceae | Phaeocryptopus | Phaeocryptopus | gaeumannii |
| Ascomycota | Sordariomycetes | Hypocreales | Clavicipitaceae | Claviceps | Claviceps | purpurea |
| Chytridiomycota | Spizellomycetes | Spizellomycetales | Spizellomycetaceae | Kochiomyces | NA | NA |
| Ascomycota | Taphrinomycetes | Taphrinales | Taphrinaceae | Taphrina | Taphrina | carpini |
| Ascomycota | Leotiomycetes | Helotiales | Hemiphacidiaceae | Rhabdocline | NA | NA |
| Ascomycota | Dothideomycetes | Capnodiales | Mycosphaerellaceae | Ramularia | Ramularia | agrimoniae |
| Ascomycota | Sordariomycetes | Xylariales | Cryptostroma | Cryptostroma | NA | NA |
| Ascomycota | Sordariomycetes | Xylariales | Diatrypaceae | Diatrype | NA | NA |
| Ascomycota | Dothideomycetes | Botryosphaeriales | Botryosphaeriaceae | Dothiorella | Dothiorella | californica |
| Ascomycota | Sordariomycetes | Magnaporthales | Magnaporthaceae | Pseudophialophora | Pseudophialophora | angusta |
| Ascomycota | Sordariomycetes | Diaporthales | Gnomoniaceae | Ophiognomonia | Ophiognomonia | alni |
| Ascomycota | Dothideomycetes | Botryosphaeriales | Botryosphaeriaceae | Dothiorella | NA | NA |
| Ascomycota | Dothideomycetes | Capnodiales | Teratosphaeriaceae | Devriesia | NA | NA |
| Ascomycota | Dothideomycetes | Pleosporales | Pleosporaceae | Curvularia | Curvularia | trifolii |
| Ascomycota | Dothideomycetes | Capnodiales | Mycosphaerellaceae | Pseudocercospora | NA | NA |
| Basidiomycota | Agaricomycetes | Cantharellales | Ceratobasidiaceae | Ceratobasidium | Ceratobasidium | cereale |
| Ascomycota | Dothideomycetes | Pleosporales | Thyrostroma | NA | NA | NA |
| Ascomycota | Sordariomycetes | Togniniales | Togniniaceae | Phaeoacremonium | NA | NA |
| Chytridiomycota | Rhizophydiomycetes | Rhizophydiales | Rhizophydiaceae | Rhizophydium | Rhizophydium | brooksianum |
| Ascomycota | Dothideomycetes | Botryosphaeriales | Botryosphaeriaceae | Lasiodiplodia | NA | NA |
| Basidiomycota | Agaricomycetes | Cantharellales | Ceratobasidiaceae | Thanatephorus | Thanatephorus | cucumeris |
| Ascomycota | Leotiomycetes | Erysiphales | Erysiphaceae | Sawadaea | Sawadaea | bicornis |
| Ascomycota | Dothideomycetes | Pleosporales | Chalastospora | Chalastospora | NA | NA |
| Basidiomycota | Ustilaginomycetes | Ustilaginales | Ustilaginaceae | Ustilago | Ustilago | hordei |
| Ascomycota | Eurotiomycetes | Coryneliales | Coryneliaceae | Caliciopsis | Caliciopsis | pinea |
| Ascomycota | Dothideomycetes | Capnodiales | Mycosphaerellaceae | Ramularia | Ramularia | collo |
| Ascomycota | Dothideomycetes | Myriangiales | Elsinoaceae | NA | NA | NA |
